# Supplementary material for: Development of a Kit for Rapid Immunochromatographic Detection of Sacbrood Virus Infecting Apis cerana (AcSBV) Based on Polyclonal and Monoclonal Antibodies Raised against Recombinant VP1 and VP2 Expressed in Escherichia coli
Source: Viruses. 2021 Dec 4;13(12):2439. doi: 10.3390/v13122439 (PMC8707083; doi:10.3390/v13122439)
Supplement: Supplementary file 1 [file viruses-13-02439-s001.zip › viruses-1469338-supplementary revised.pdf]

Supplementary Materials

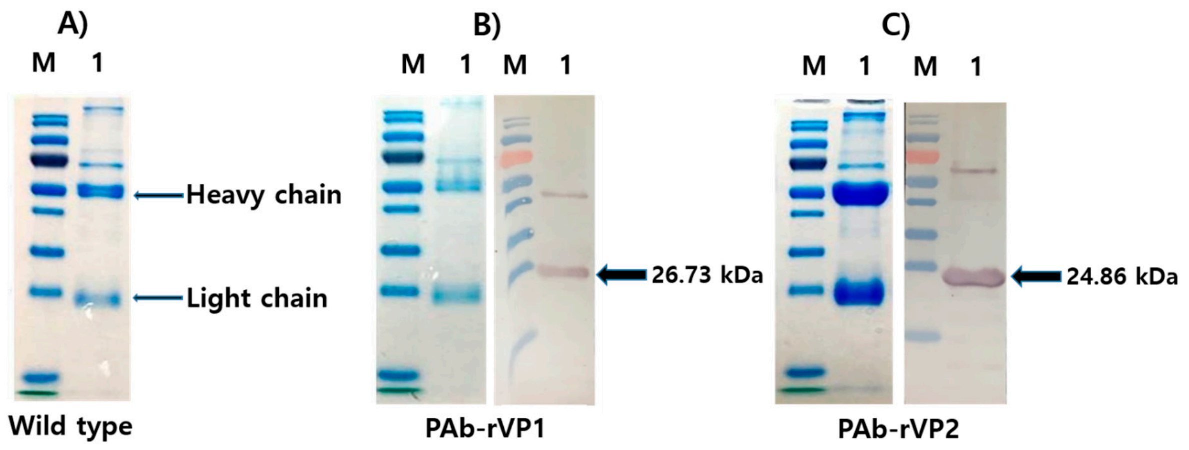

Figure S1. (A) 10% SDS-PAGE of the purified IgG from blood collected from wild type mouse at the 7th week. (B) 10% SDS-PAGE of the purified IgG (PAb-rVP1) from antiserum collected from rVP1-inmunized mouse at 7th week (left) and Western blot analysis of the rVP1 using PAb-rVP1 (right). (C) 10% SDS-PAGE of the purified IgG (PAb-rVP2) from antiserum collected from rVP2-inmunized mouse at 7th week (left) and Western blot analysis of the rVP2 using PAb-rVP2 (right).

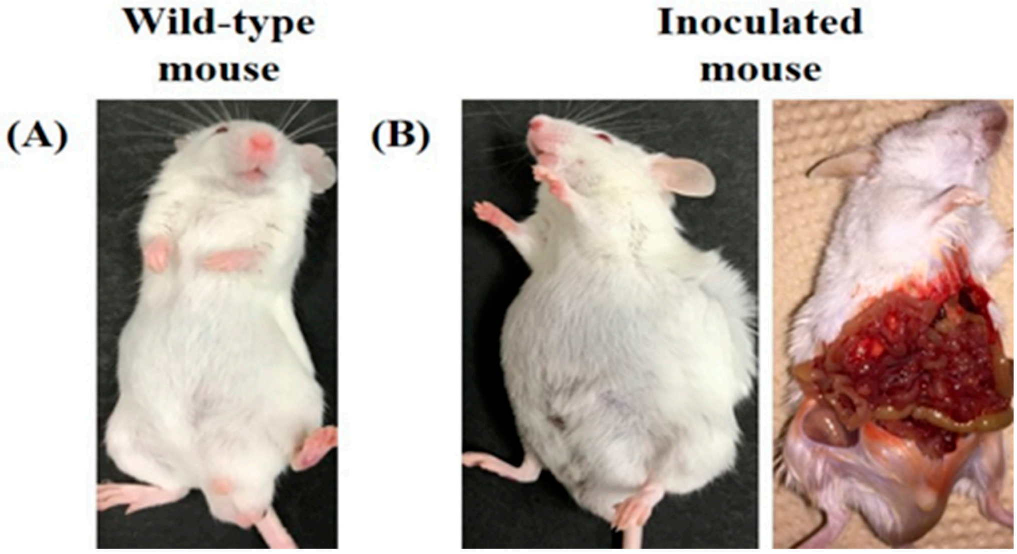

Figure S2. Mouse intraperitoneal injected with hybridoma cells containing mAb-VP1 and mAb-VP2, respectively. Ascites formation and monoclonal antibody production was carried out in Freund's incomplete adjuvant-primed male mice (6-10 weeks old). Ascites fluid containing a high level of antibody was collected from each mouse at 10 days after inoculation. The animals usually survived 11–16 days after being injected with the tumor cells.

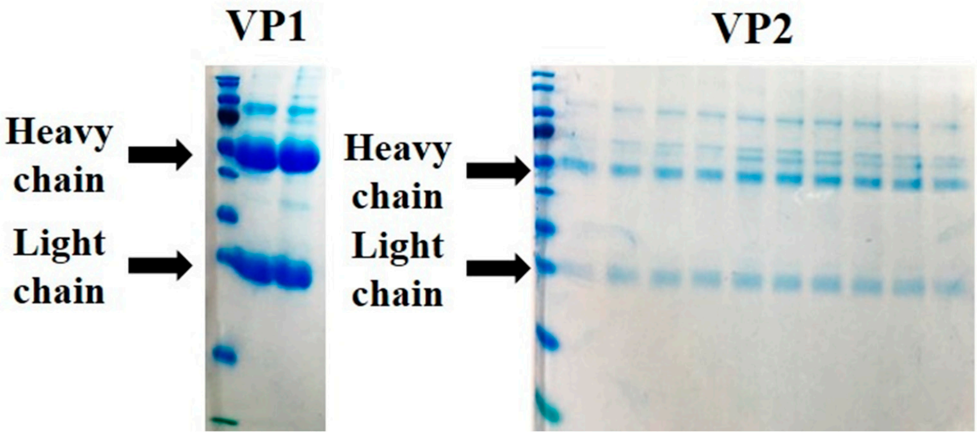

Figure S3. Purified monoclonal IgG-VP1 and -VP2 from ascites of mice which were injected with hybridoma cells containing mAb-VP1 and mAb-VP2, respectively.

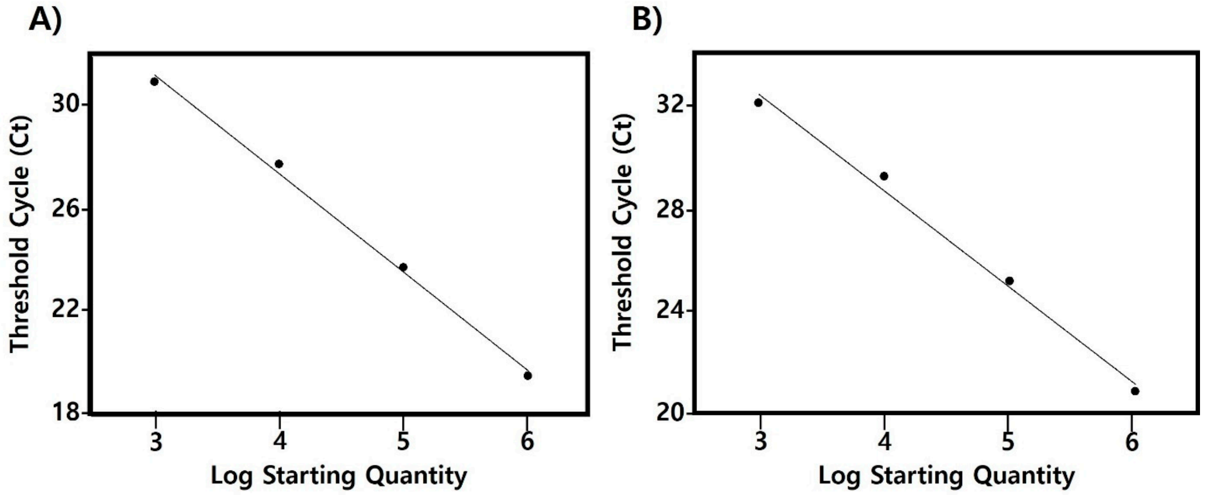

Figure S4. A ten-fold serial dilution series of the pET28a-VP1 and pET28a-VP2 ranging from 1×10^3 to 1×10^6 copies/μL, was used to construct the standard curves for both VP1 andVP2. Each standard dilution was amplified by real-time qPCR using primer sets of VP1 and VP2 in duplicate. The plot of a standard curve of Ct values against the logarithmic dilutions produced a regression line ($y = -3.8356x + 42.7930$, $R^2 = 0.9965$) for the VP1 (A) and a regression line ($y = -3.7314x + 43.5159$, $R^2 = 0.9933$) for the VP2 (B).

No.	Real-time RT-PCR		mAb-VP1-1	
	Data	Copy number	Reaction	Results
1	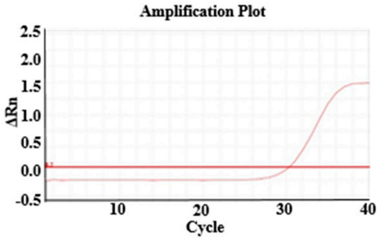	(3.02×10^{3}copies)	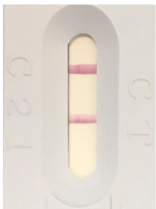	positive
2	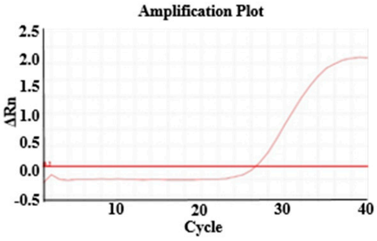	(4.33×10^{4}copies)	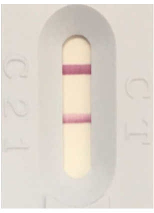	positive
3	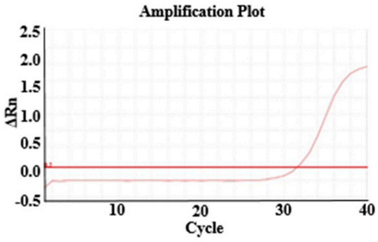	(1.38×10^{3}copies)	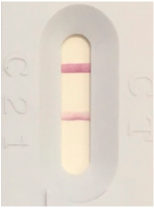	positive
4	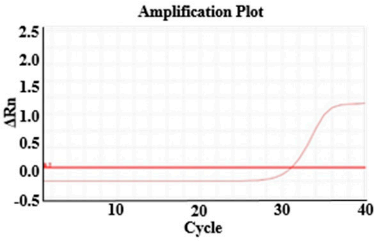	(1.92×10^{3}copies)	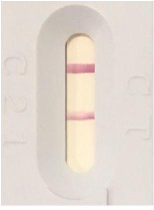	positive
5	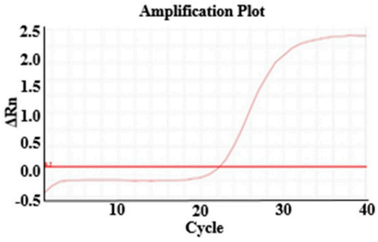	(8.39×10^{5}copies)	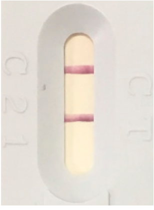	positive
6	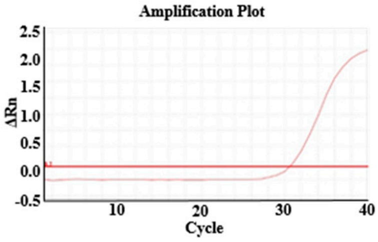	(2.62×10^{3}copies)	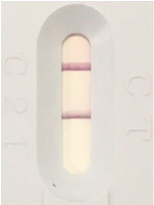	positive

7	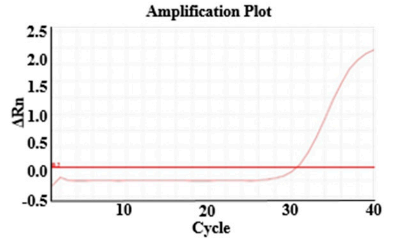	(2.53×10^{3}copies)	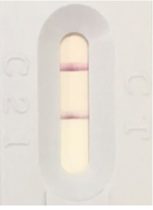	positive
8	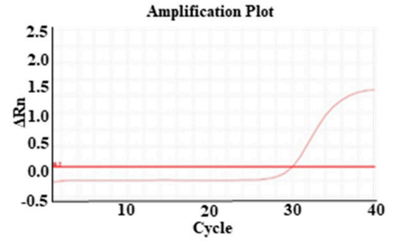	(5.86×10^{3}copies)	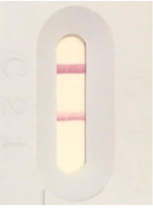	positive
9	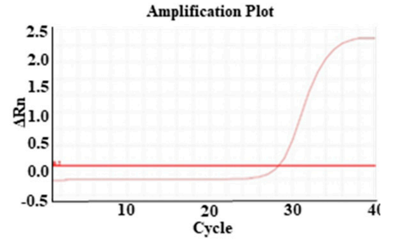	(1.29×10^{4}copies)	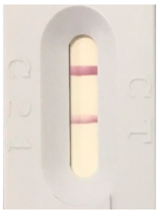	positive
10	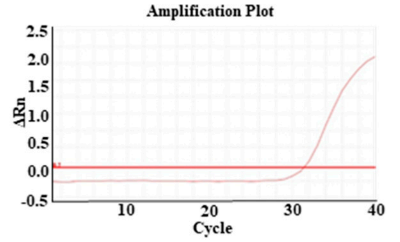	(1.54×10^{3}copies)	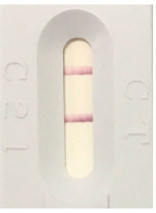	positive
11	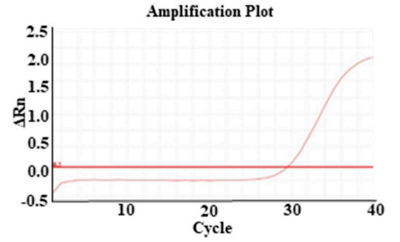	(5.7×10^{3}copies)	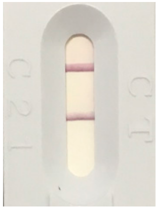	positive
12	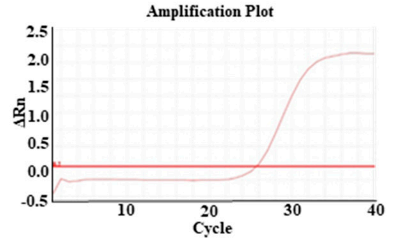	(7.78×10^{4}copies)	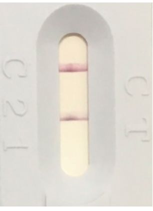	positive
13	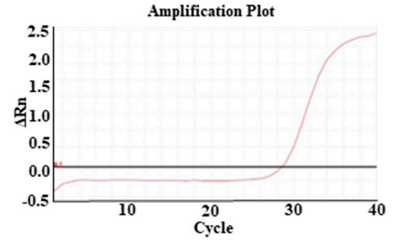	(1.08×10^{4}copies)	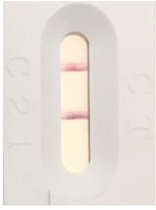	positive

14	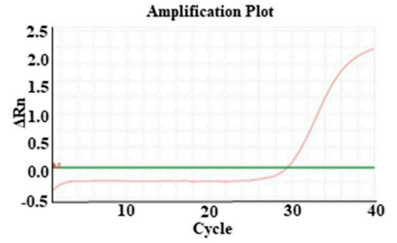	(5.86×10^3copies)	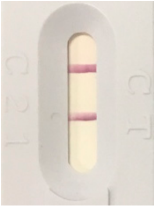	positive
15	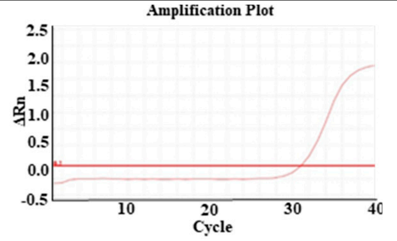	(1.97×10^3copies)	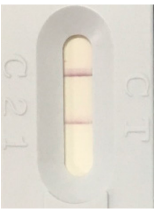	positive
16	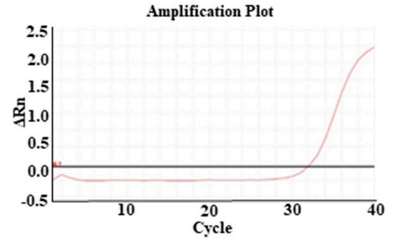	(1.04×10^3copies)	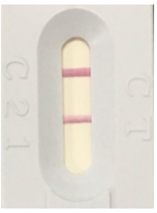	positive
17	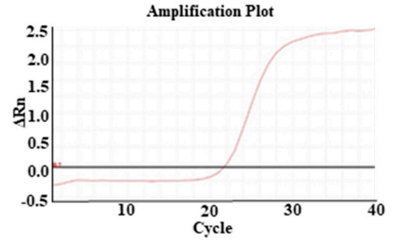	(1.22×10^6copies)	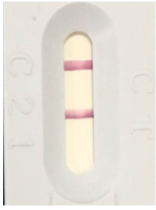	positive
18	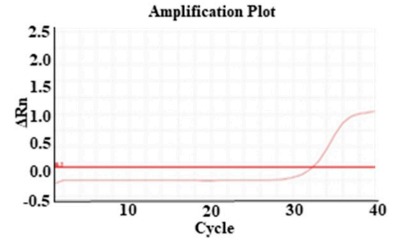	(0.81×10^3copies)	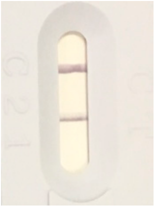	positive
19	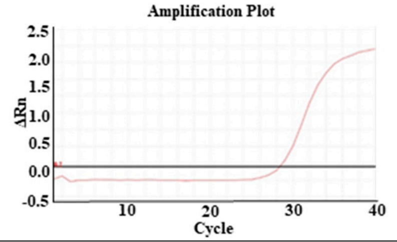	(1.21×10^4copies)	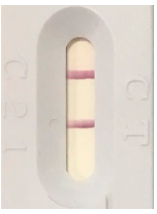	positive
20	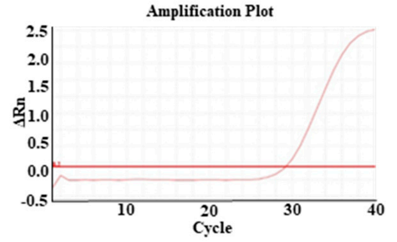	(6.77×10^3copies)	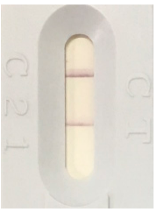	positive

21	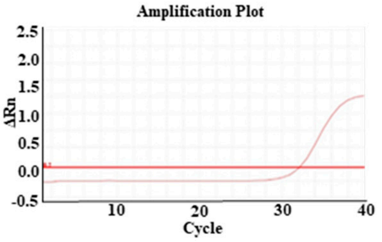	(0.97×10^{3}copies)	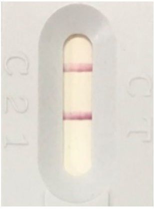	positive
22	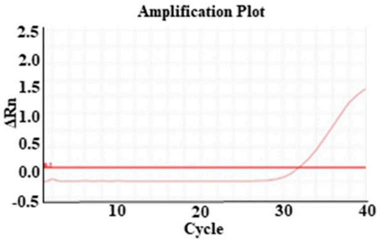	(1.03×10^{3}copies)	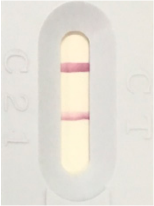	positive
23	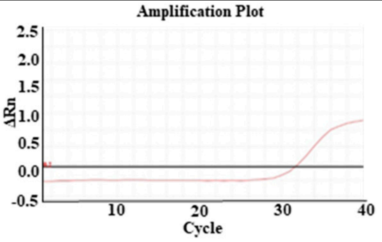	(1.28×10^{3}copies)	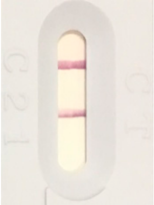	positive
24	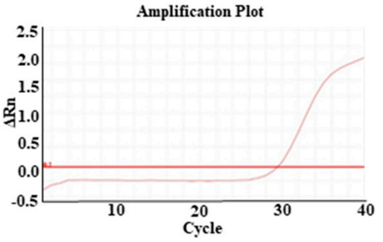	(6.07×10^{3}copies)	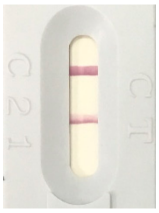	positive
25	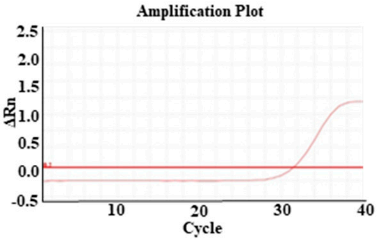	(1.42×10^{3}copies)	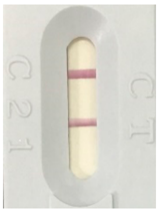	positive
26	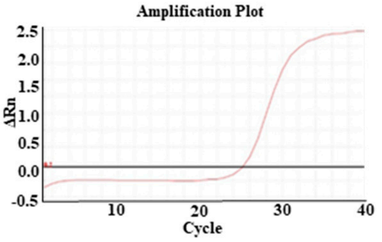	(1.17×10^{5}copies)	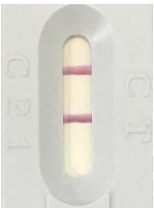	positive
27	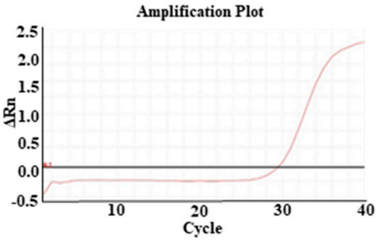	(5.74×10^{3}copies)	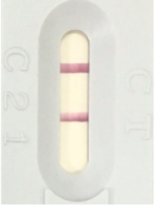	positive

28	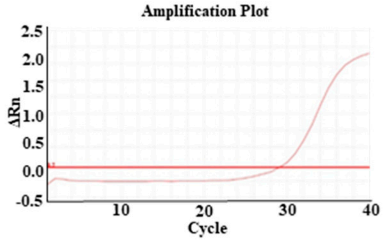	$(7.23 \times 10^3$copies)	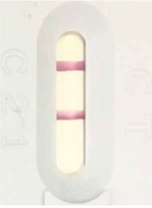	positive
29	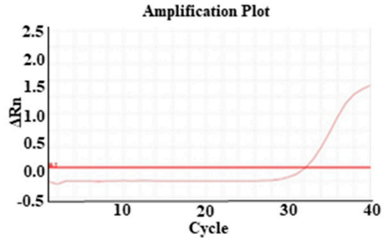	$(0.95 \times 10^3$copies)	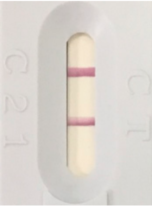	positive
30	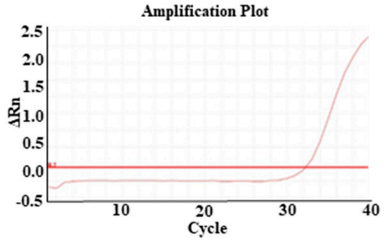	$(0.86 \times 10^3$copies)	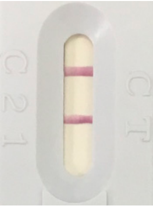	positive
31	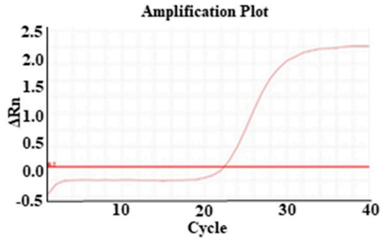	$(7.62 \times 10^5$copies)	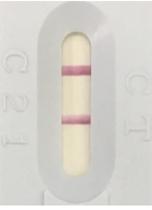	positive
32	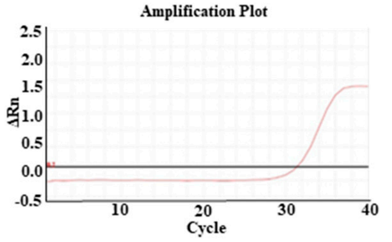	$(1.58 \times 10^3$copies)	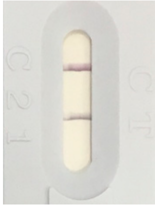	positive
33	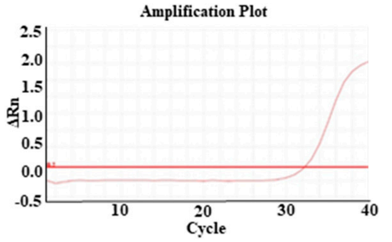	$(0.86 \times 10^3$copies)	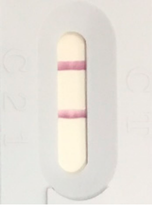	positive
34	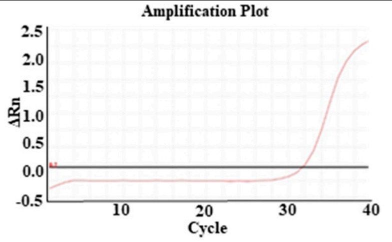	$(1.2 \times 10^3$copies)	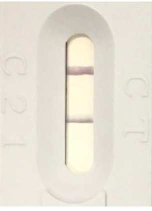	positive

35	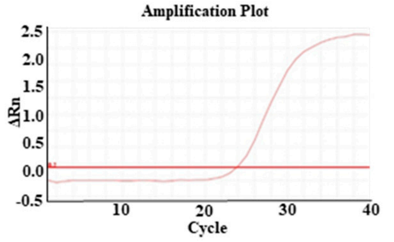	(2.71×10^{5}copies)	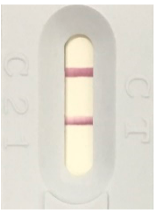	positive
36	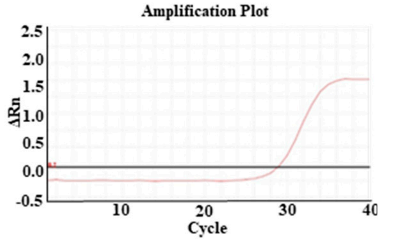	(8.56×10^{3}copies)	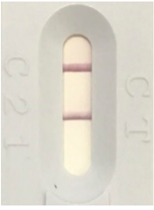	positive
37	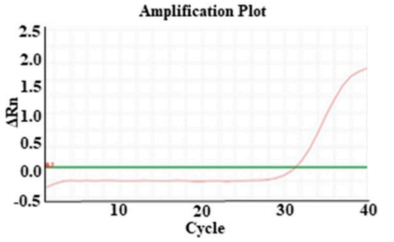	(1.65×10^{3}copies)	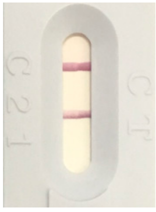	positive
38	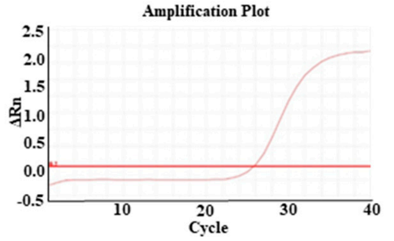	(7.31×10^{4}copies)	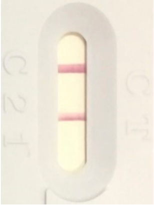	positive
39	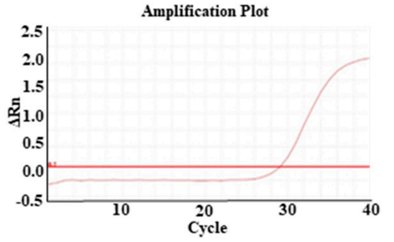	(7.72×10^{3}copies)	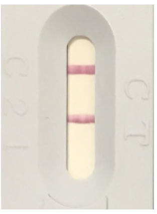	positive
40	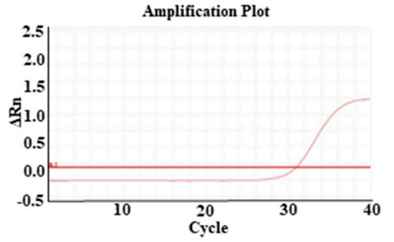	(2.07×10^{3}copies)	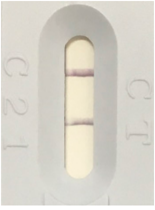	positive
41	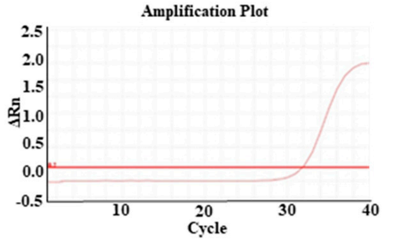	(1.1×10^{3}copies)	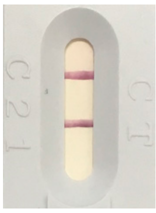	positive

42	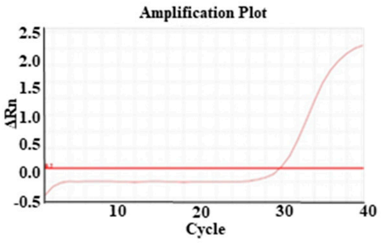	(4.59×10^3copies)	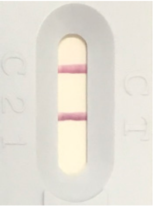	positive
43	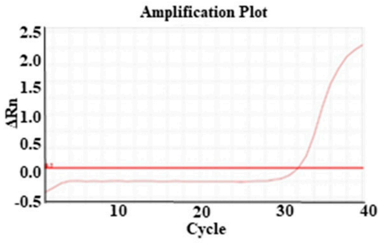	(9.83×10^2copies)	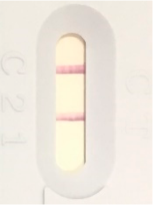	positive
44	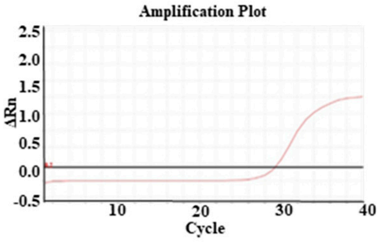	(7.01×10^3copies)	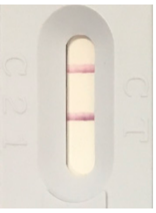	positive
45	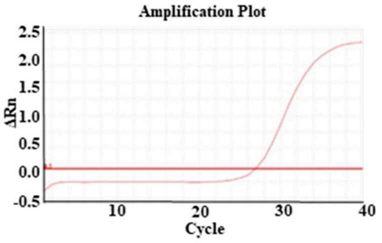	(3.26×10^4copies)	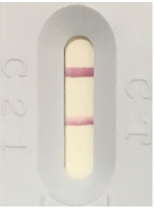	positive
46	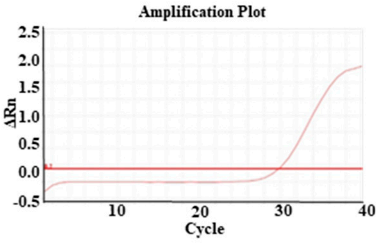	(4.48×10^3copies)	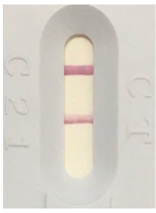	positive
47	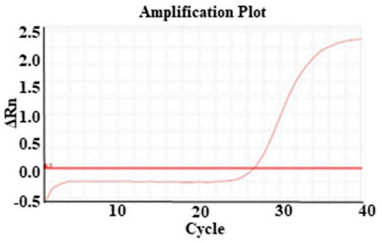	(3.72×10^4copies)	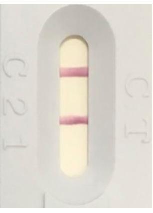	positive

<table>
<tr><td>48</td><td>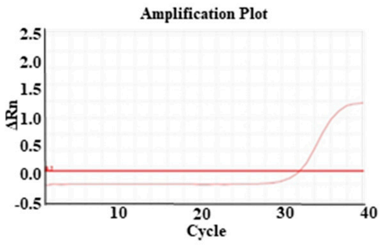
</td><td>(0.9×10^{3}copies)</td><td>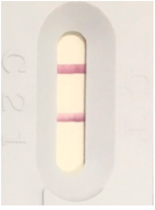</td><td>positive</td></tr>
<tr><td>49</td><td>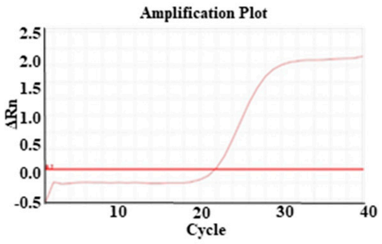
</td><td>(1.12×10^{6}copies)</td><td>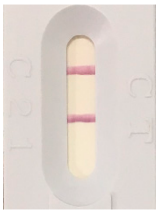</td><td>positive</td></tr>
<tr><td>50</td><td>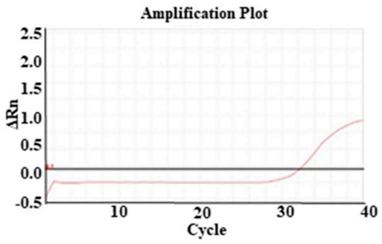
</td><td>(0.92×10^{3}copies)</td><td>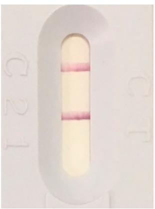</td><td>positive</td></tr>
</table>

Figure S5. Sensitivity test of mAb-V1-1.

No.	Real-time RT-PCR		mAb-VP1-1	
	Data	Copy number	Reaction	Results
1	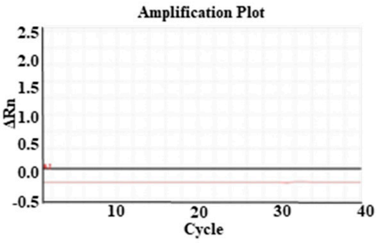	0	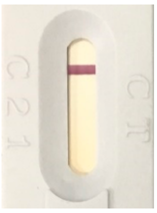	negative
2	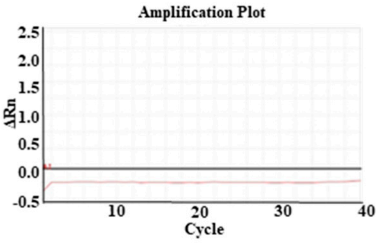	0	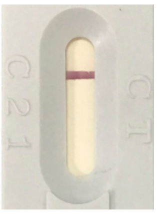	negative
3	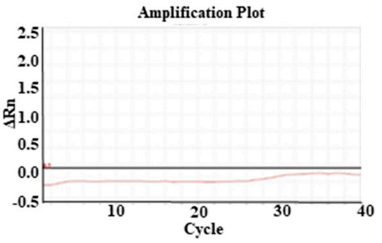	0	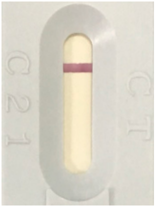	negative
4	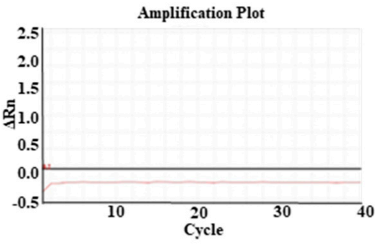	0	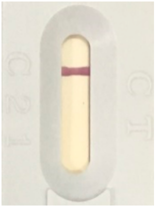	negative
5	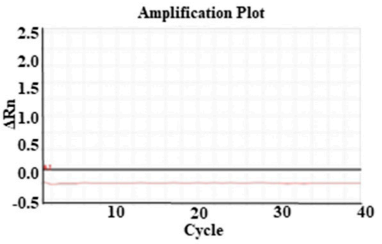	0	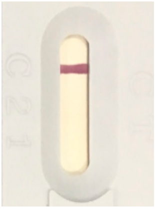	negative

6	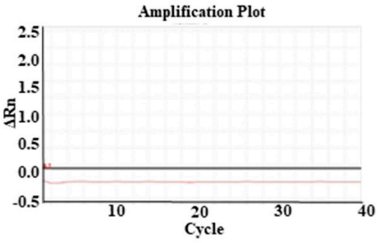	0	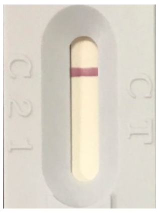	negative
7	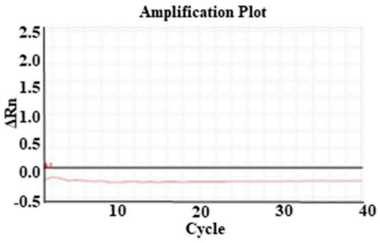	0	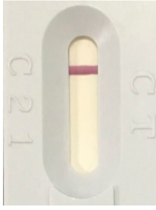	negative
8	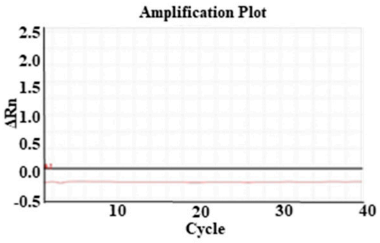	0	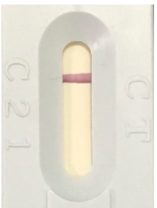	negative
9	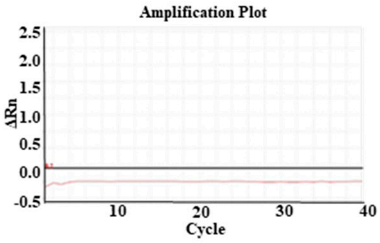	0	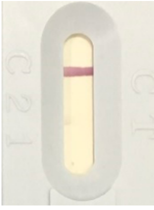	negative
10	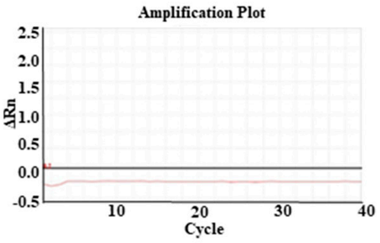	0	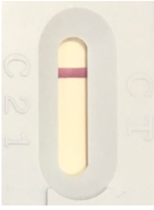	negative
11	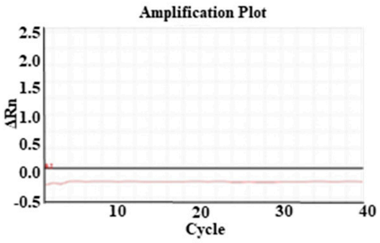	0	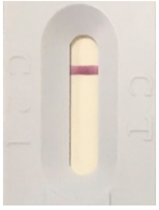	negative

12	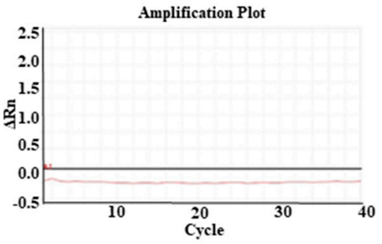	0	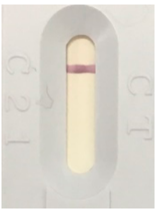	negative
13	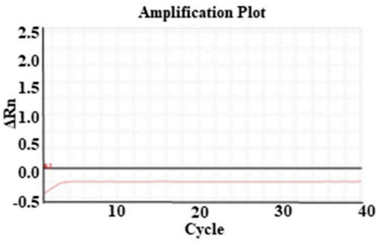	0	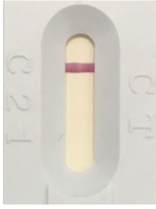	negative
14	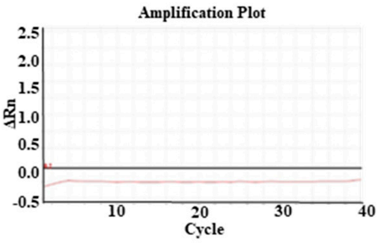	0	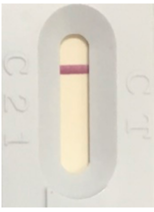	negative
15	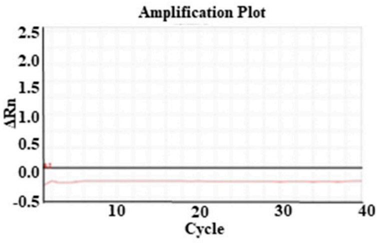	0	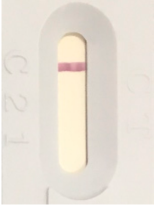	negative
16	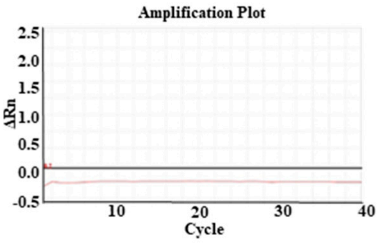	0	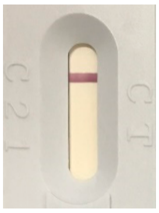	negative
17	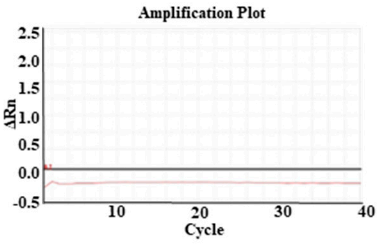	0	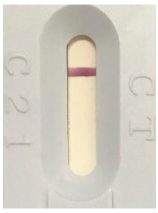	negative

18	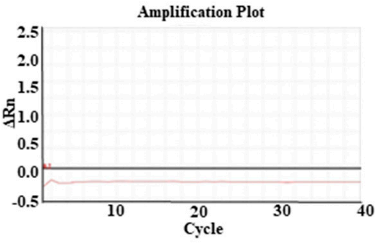	0	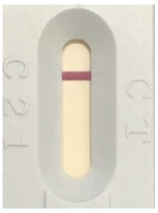	negative
19	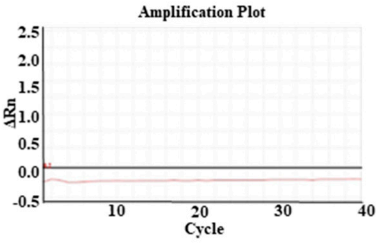	0	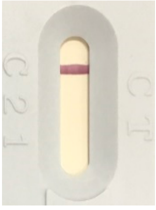	negative
20	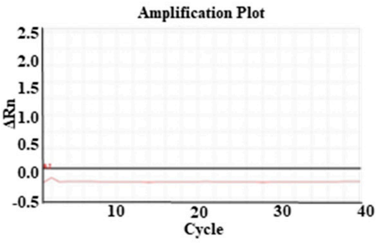	0	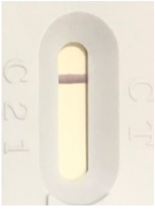	negative
21	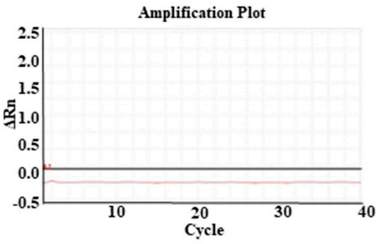	0	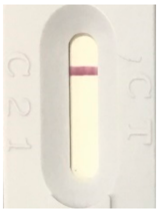	negative
22	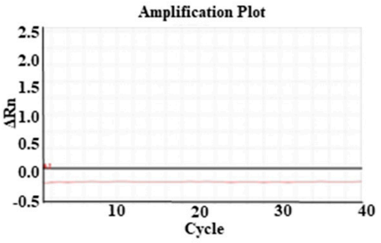	0	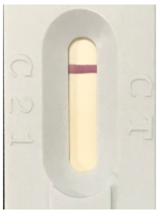	negative
23	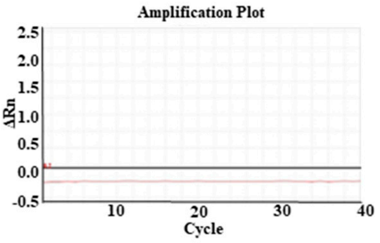	0	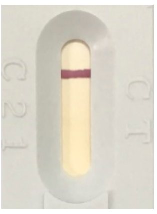	negative

24	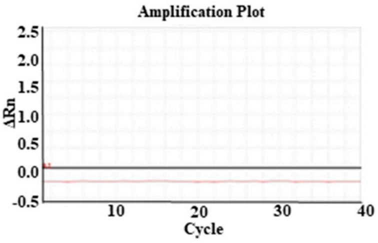	0	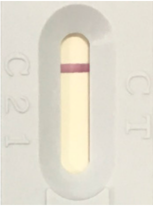	negative
25	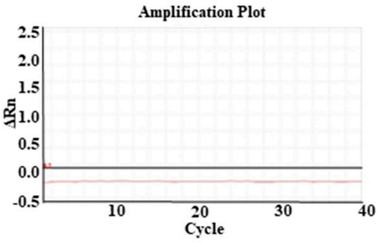	0	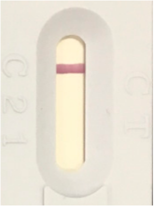	negative
26	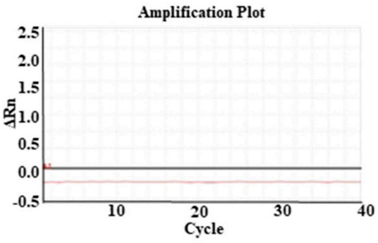	0	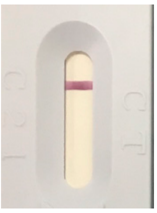	negative
27	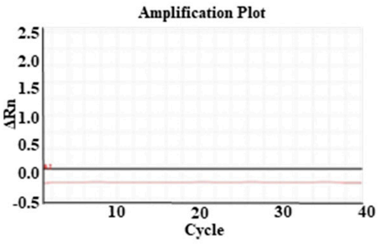	0	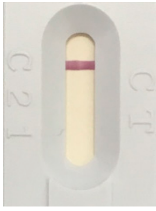	negative
28	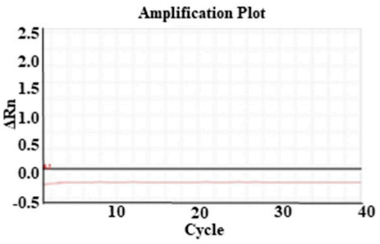	0	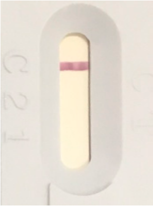	negative
29	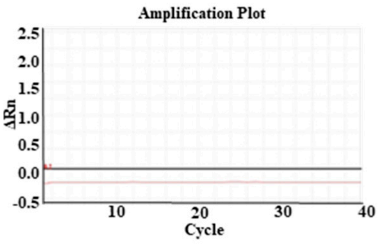	0	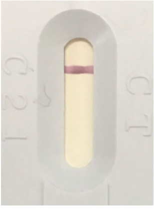	negative

30	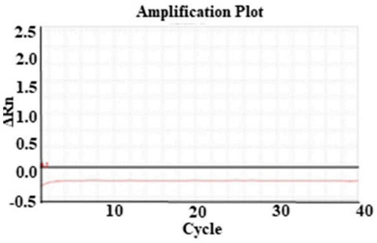	0	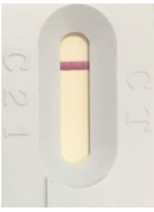	negative
31	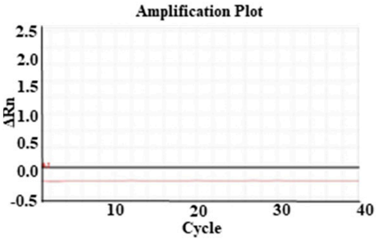	0	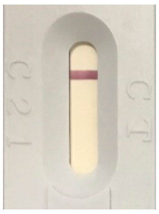	negative
32	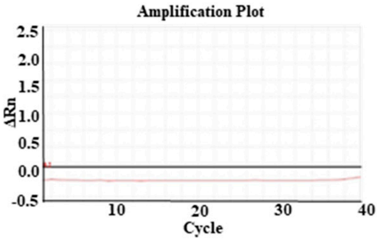	0	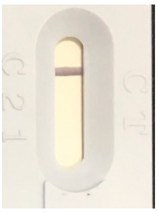	negative
33	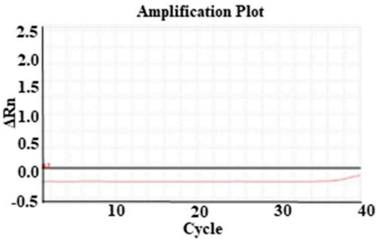	0	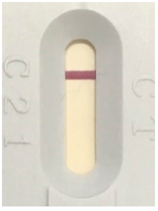	negative
34	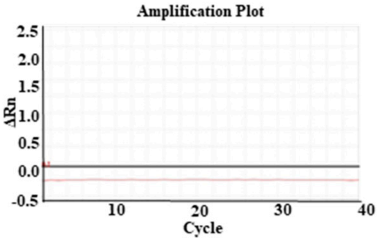	0	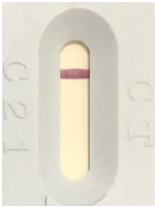	negative
35	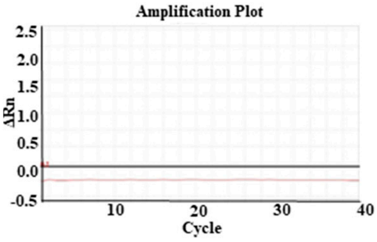	0	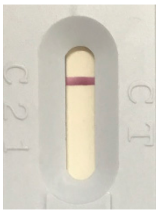	negative

36	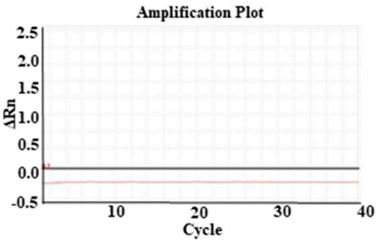	0	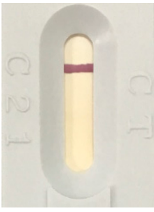	negative
37	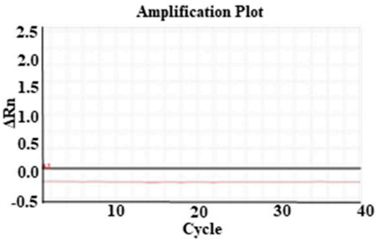	0	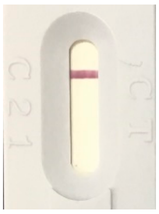	negative
38	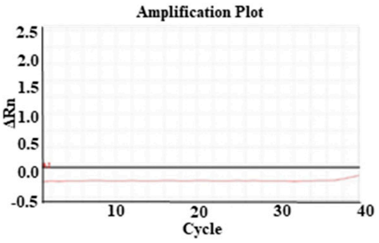	0	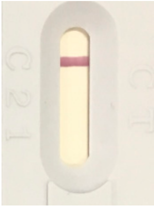	negative
39	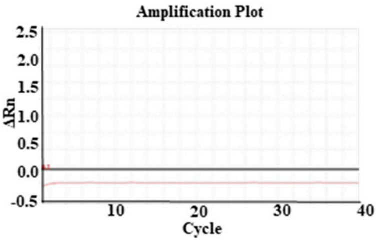	0	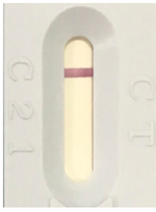	negative
40	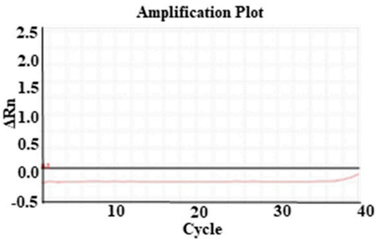	0	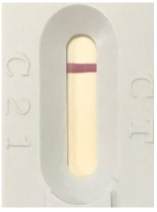	negative
41	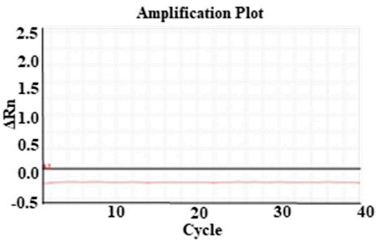	0	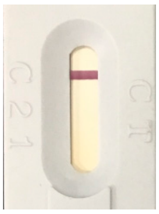	negative

42	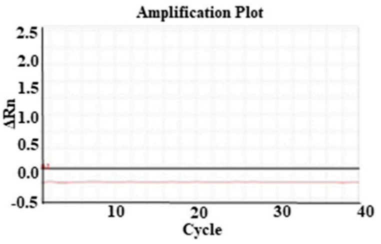	0	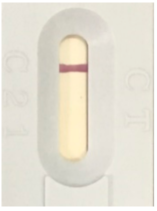	negative
43	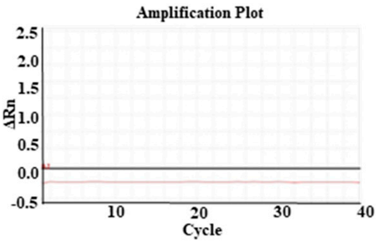	0	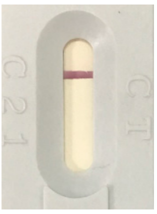	negative
44	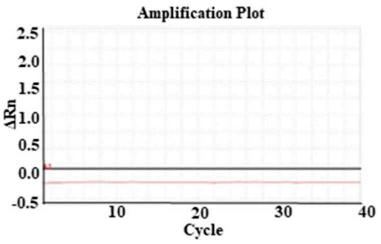	0	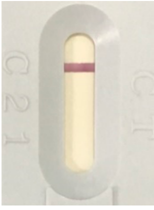	negative
45	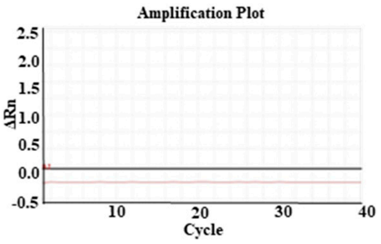	0	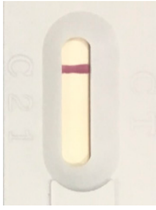	negative
46	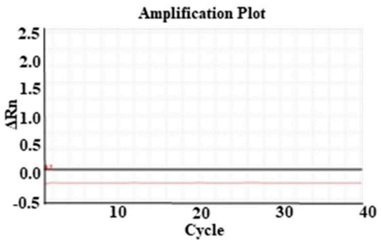	0	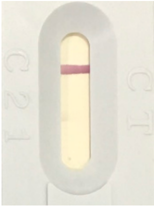	negative
47	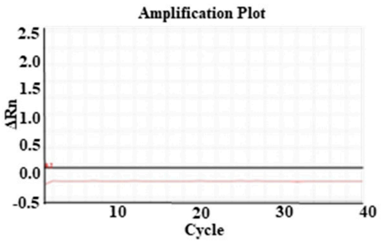	0	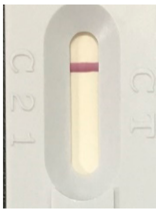	negative

48	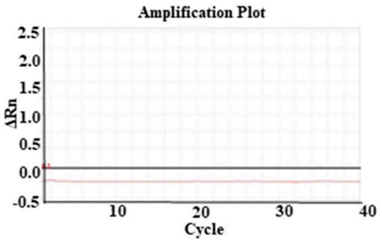	0	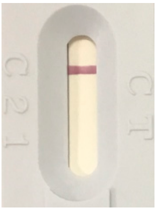	negative
49	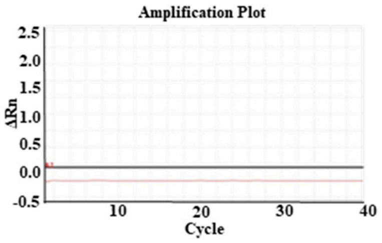	0	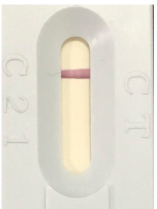	negative
50	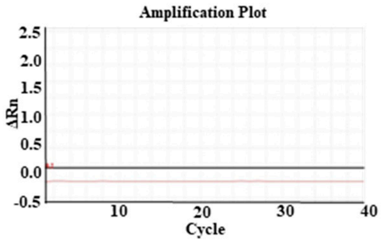	0	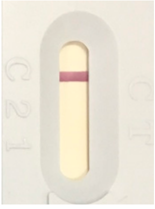	negative

Figure S6. Specificity test of mAb-V1-1.
